# Urgent-Start Peritoneal Dialysis as a Bridge to Definitive Chronic Renal Replacement Therapy: Short- and Long-Term Outcomes

**DOI:** 10.3389/fphys.2018.01830

**Published:** 2019-01-04

**Authors:** Ewa Wojtaszek, Agnieszka Grzejszczak, Katarzyna Grygiel, Jolanta Małyszko, Joanna Matuszkiewicz-Rowińska

**Affiliations:** ^1^Department of Nephrology, Dialysis and Internal Medicine, Warsaw Medical University, Warsaw, Poland; ^2^Department of General, Vascular and Transplant Surgery, Warsaw Medical University, Warsaw, Poland

**Keywords:** peritoneal dialysis, short-term outcomes, mechanical complication, technique survival, patient survival, long-term outcomes, infectious complications

## Abstract

**Background:** The peritoneal dialysis (PD) urgent-start pathway, without typical 2-week break-in period, was meant for late-referral patients able and prone to join PD-first program, with its main advantages such as: keeping the vascular system intact, preserving their residual renal function and retaining life-style flexibility. We compared the short- and long-term outcomes of consecutive 35 patients after urgent- and 94 patients after the planned start of PD as the first choice.

**Methods:** The study included all incident end-stage renal disease patients starting PD program between January 2005 and December 2015, classified into two groups: those with urgent (unplanned) and those with elective (planned) start. Urgent PD was initiated as an overnight automatic procedure (APD) with dwell volume gradually increased, and after 2–3 weeks, target PD method was established.

**Results:** The mean time between catheter implantation and PD start was 3.5 ± 2.3 in urgent and 16.2 ± 1.7 days in planned-start groups (*p* < 0.00001). 51% of the patients in the urgent-start group required PD during first 48 h after catheter insertion. Mean follow-up of 17.6 ± 11.09 months (median: 19.0) was in the urgent-start group and 28.6 ± 26.6 months (median: 19.5) in the planned-start group. The early mechanical complications were observed more often in the urgent-start group (29 vs. 4%, *p* = 0.00005). The only significant predictors of early mechanical complications were serum albumin (*p* = 0.02) and time between the catheter insertion and PD start. The first year patient survival and technique survival censored for death and kidney transplantation were not significantly different between groups. In Cox proportional analysis the independent risk factors for patient survival as well as for method and patient survival appeared Charlson Comorbidity Index CCI (HR 1.4; *p* = 0.01 and 1.24; *p* = 0.02) and time from catheter implantation to PD start with HR 5.11; *p* = 0.03 and 4.29; *p* = 0.04 for <2 days, while time >14 days lost its predictive value (*p* = 0.07).

**Conclusion:** Peritoneal dialysis may be a feasible and safe alternative to HD in patients who need to start dialysis urgently without established dialysis access, with an acceptable complications rates, as well as patient and technique survival.

## Introduction

Almost 3 million people worldwide are affected by end stage renal disease (ESRD), and the vast majority of those who cannot undergo renal transplantation are treated with hemodialysis (HD), while less than 10% are on peritoneal dialysis (PD) (Liyanage et al., 2015; Li and Kwong, 2017). Moreover, despite many advantages, comparable survival rates for both techniques and the efforts of PD societies, the numbers of PD patients are still declining, year by year (Mehrotra et al., 2016; Li et al., 2017). The problem is complex, with the main causes being: an inadequate education and training of the nephrologists, shortages of trained nurses, easy access to HD, lack of patients education, and, in some countries, financial issues. Another important aspect is that as much as 40–60% of the patients initiate dialysis urgently, with no dialysis access, due to a late referral (Perl et al., 2011; Blake et al., 2013).

Since the insertion of central venous catheter is readily available and much easier than PD catheter placement, HD becomes the typical initial modality in such situations and many remain on HD, taking the path of least resistance. This can be overcome by introducing a so-called PD urgent-start program, which has attracted increased interest in recent years (Povlsen and Ivarsen, 2006; Lobbedez et al., 2008; Sharma et al., 2008; Oliver et al., 2012; Arramreddy et al., 2014; Mahnensmith, 2014).

A program of this kind was introduced in the Department of Nephrology of the Medical University of Warsaw in 2004. The aim of our study was to compare the impact of planned and unplanned (urgent) initiation of PD therapy in ESRD patients on infectious and non-infectious complications rate, and patients as well as patient technique short- and long-term survival.

## Materials and Methods

All incident patients with ESRD starting a PD program in the Department of Nephrology at the Medical University of Warsaw between January 2005 and December 2015 were enrolled into the study. The protocol was approved by the Bioethical Committee of Medical University of Warsaw. All patients gave written informed consent in accordance with the Declaration of Helsinki.

In accordance with the International Society for PD (ISPD) (Li et al., 2016) and the European Renal Best Practice (ERBP) guidelines (Dombros et al., 2005), they were classified into two groups: those with urgent (unplanned) and those with elective (planned) start, depending on whether PD initiation took place during less than 14 days after peritoneal access creation or later.

In every late referral case the decision to initiate PD urgently was made individually in three steps. First, in patients with life-threatening metabolic disturbances (hyperkalemia, severe acidosis, severe volume overload, or marked uremia) the PD catheter implantation was preceded by 1–3 emergency HD procedures performed via temporary femoral vein access. Secondly, any medical or social contradictions to PD were carefully checked. In the last step, eligible patients were offered an informed choice of dialysis modality.

In those who opted for PD, a Tenckhoff catheter (straight or coiled) was inserted by open surgery under local or general anesthesia. PD was initiated as an overnight automatic procedure (APD) in supine position with 800–1000 mL dwell volume (for patients <, and ≥ 60 kg, respectively) and a dry day. The dwell volumes were gradually increased, and after 2–3 weeks patients remained on standard APD or were converted to continuous ambulatory PD (CAPD).

Follow up analyses of mechanical (leak, hernia, catheter migration, catheter obstruction, and bleeding) and infectious (peritonitis, exit site/tunnel infection) complications were performed after first 4 weeks, 90 days, and 12 months. Patients and technique survival rates were evaluated after first 90 days, 12 months, and at the end of the observation. The patients were observed for 3306 patient-moths (for entire group mean 25.6 ± 23.8, median 19 months); for planned-start group 28.6 ± 26.6, median 19.5 months, for urgent-start group 17.6 ± 0.9, median 19.0 months. Peritonitis and ESI/TI rates are expressed as episodes per year of treatment. Catheter migration as reflected by clinical suspicion was defined as lack/drop of ultrafiltration or problems with dialysate outflow. This suspicion was confirmed by X-ray of abdominal cavity; Catheter obstruction did not occur in our patients. Pericatheter leak (exit-site leak) was defined as fluid leak around the catheter exit-site. Abdominal leak on clinical suspicion seen as drop of ultrafiltration, asymmetric swelling of abdominal wall tissues and “orange peel” syndrome; was confirmed on radiological examination with contrast given to the catheter or during CT.

### Statistical Analysis

Statistical analysis was performed with STATISTICA software package (version12), StatSoft Poland. Continuous variables with normal distribution were presented as mean (standard deviation [SD]) and compared between two groups using Student’s *t*-test. Non-normal variables were expressed as median (interquartile range [IQR]) and compared with Mann–Whitney *U*-test. Categorical variables were presented as frequencies and percentages, and compared using Chi^2^ test. The survival rates were analyzed using the Kaplan–Meier technique and log-rank test. The multivariate Cox proportional hazards model was used in survival analysis to adjust outcomes for confounding variables such as: estimated glomerular filtration rate (eGFR), serum albumin and blood hemoglobin concentration, Charlson Comorbidity Index (CCI), time between catheter insertion and PD start, any complication appearance during first 4 weeks of the treatment and the urgent start *per se.* The statistical difference was considered to be significant for *p* < 0.05.

## Results

### Patient Characteristics

One hundred twenty nine incident patients (56% men), aged 50.8 ± 17.8 years entered the study. Among them, 35 (27%) started PD urgently and the remaining 94 (73%) in the planned manner. The main demographic and clinical data of the unplanned and planned beginners are presented in Table 1. There were no significant differences between study groups in terms of age, sex, and CCI. The patients who started PD urgently had worse kidney function, lower serum albumin, and blood hemoglobin concentrations at the beginning of the treatment.

**Table 1:** The main demographic and clinical characteristics of the study groups.

Parameter	Urgent-start (*n* = 35)	Planned-start (*n* = 94)	*p*
Age (*years*)	51 ± 18.5	51 ± 17.7	NS
Sex (*% of men*)	49	52	NS
eGFR (*ml/min/1.73 m^2^*)	6.1 ± 2.9	8.1 ± 2.7	<0.001
Serum albumin (*g/dl*)	3.23 ± 0.36	3.55 ± 0.46	<0.01
Blood hemoglobin (*g/dl*)	9.3 ± 1.36	10.4 ± 1.27	<0.0001
Diabetes (%)	11	36	<0.0001
Cause of kidney disease (%)			<0.01
∙ diabetic kidney disease	9	25	
∙ glomerulonephritis	37	43	
∙ hypertensive nephropathy	9	11	
∙ unknown/other	45	21	
Charlson comorbidity index (CCI)	6 ± 3	5 ± 3	NS


### Dialysis Initiation

The mean time between peritoneal access creation and PD start was 3.5 ± 2.3 and 16.2 ± 1.7 days, in urgent- and planned-start groups, respectively (*p* < 0.00001). Fifty-one percent of patients in the urgent-start group required PD during first 48 h after catheter insertion, and in 3 of them with life-threatening clinical symptoms – 2–3 short emergency HD procedures via femoral access were performed before PD catheter placement. In the remaining patients who started the treatment urgently, the time to PD commencement ranged between 3 and 8 days.

### Non-infectious Complications

All mechanical complications are presented in Table 2. The early ones were observed more often in the urgent-start group (29% of patients vs. 4%, *p* = 0.00005), however, with no need for surgical intervention or temporary transition to HD. The most frequent early complication was dialysate leakage, which occurred in four patients – all of them started PD within 48 h after catheter implementation. In these patients, PD was postponed for 3–5 days and subsequently resumed with low-volume dwells.

**Table 2:** Rate of early (first 4 weeks) and late (>4 weeks) mechanical complications in the studied groups.

Complication	Urgent-start (*n* = 35)		Planned-start (*n* = 94)		*p*	
	Early	Late	Early	Late	Early	Late
Leakage	4 (11%)	5 (14%)	0	7 (7%)	<0.001	NS
Bleeding	3 (9%)	0	1 (1%)	0	<0.05	–
Catheter migration	3 (9%)	1 (3%)	3 (3%)	15 (16%)	NS	0.04
Catheter obstruction	0	0	0	0	–	–
Hernia	0	2 (6%)	0	12 (13%)	–	NS


The late mechanical complications occurred in 33.5% of all studied patients, 20% in urgent-start and 31% in the planned-start group (*p* = 0.15). The rate of late non-infectious complications was similar in both groups, with the exception of PD catheter migration, more frequently seen in the planned-start group.

In the regression logistic analysis, serum albumin as continuous variable (0.18; CI: 0.04–0.77; *p* = 0.02) and time between the catheter insertion and PD start expressed as categorical variables: ≤48 h (1,79; CI: 0,45–7,18; *p* = 0,02), and >14 days (0.08; CI: 0.01–0.5; *p* = 0.003) occurred the only significant predictors of early mechanical complications. In regard to long-term follow-up, neither the time between the catheter insertion and PD start, the urgent start *per se* nor any early complication influenced the occurrence of late mechanical events.

### Infectious Complications

There were no infectious complications in any studied patient during the first 4 weeks of PD treatment. During the whole observation at least one episode of peritonitis was observed—in 12 patients (34%) in the urgent-start group and in 31 (33%) patients in the planned-start group. There were no differences between the groups regarding peritonitis and/or exit-site/tunnel infection rates during the first year of PD therapy as well as during the whole observation period (Tables 3, 4). In the regression logistic analysis none of analyzed parameters had an influence on peritonitis or exit-site/tunnel infection occurrence.

**Table 3:** Peritonitis rates and time to first episode in both studies groups.

Period	Urgent-start (*n* = 35)		Planned-start (*n* = 94)		*p*	
Evaluated parameter	(A) Number of episodes/rate [episodes/year]	(B) Time to first episode (median) [months]	(A) Number of episodes/rate [episodes/year]	(B) Time to first episode (median) [months]	A	B
First year of PD	6/0.17	6.8 ± 2.6 (7)	10 /0.10	8.4 ± 2.6 (8)	NS	NS
Whole observation	14/0.40	13.9 ± 10.5 (11.5)	42 /0.44	27.5 ± 21.3 (22)	NS	0.04


**Table 4:** Exit-site/tunnel infections rates and time to first episode in both studies groups.

Period	Urgent-start (*n* = 35)		Planned-start (*n* = 94)		*p*	
Evaluated parameter	(A) Number of episodes /rate [episodes/year]	(B) Time to first episode (median) [months]	(A) Number of episodes/rate [episodes/year]	(B) Time to first episode (median) [months]	A	B
First year of PD	1/0.02	10.0 ± 2.8 (10)	6/0.06	8.3 ± 2.8 (9)	NS	NS
Whole observation	3/0.08	11.3 ± 3.1 (12)	14/0.1	23.6 ± 18.1 (14)	NS	NS


### The Technique and Patients Survival

The patients were observed for 3306 patient-months, with mean follow-up of 17.6 ± 11.09 months (median: 19.0, range: 1.0–44 months) in the urgent-start group and 28.6 ± 26.6 months (median: 19.5, range: 1.0–103 months) in the planned-start group. The short- and long-term outcomes in studied patients are presented in Table 5. The analysis revealed that PD urgent-start was associated with reduced survival but only in the first 90 days of the therapy (86 vs. 99%; *p* < 0.0001), and at the end of observation the rates were similar. During the study period five patients died of peritonitis (1 from US group and 4 from PLS group), which creates quite a high mortality rate due to PD peritonitis in our population (5/56 episodes). A review of these cases revealed: all patients had high comorbidity: CCI 7–9; all episodes occurred > 12 months from PD start; in two cases polymicrobial peritonitis was secondary to bowel perforation; in one case due to Gram negative (*E. coli*); in one case due polymicrobial (*E. coli*, *Enterococcus* fecal, *Enterobacter cloacae*, *Candida* spp.) infection without proven bowel perforation; in one case it occurred in the course of sepsis secondary to the complications after surgical treatment of lower limb ischemia. Thus, peritonitis was one of the contributing cause of death in these seriously ill patients. The causes of death are shown in Table 6.

**Table 5:** The short- and long-term outcomes in studied patients.

	Died		Receive transplant		Changed to HD		Stayed on PD		Technique survival^∗∗^	
	
90 days	6 (4.6%)		1 (0.8%)		4 (3.1%)		118 (91.5%)		118 (97%)	
	US	PLS	US	PLS	US	PLS	US	PLS	US	PLS
	5^∗^ (14%)	1 (1.1%)	0	1 (1.1%)	0	4 (4.2%)	30 (86%)	88 (94%)	30 (100%)	88 (95.7%)

90 days–12 months	8 (6%)		17 (13%)		11 (9%)		93 (72%)		93 (89%)	
	US	PLS	US	PLS	US	PLS	US	PLS	US	PLS
	6 (17%)	2 (2%)	3 (9%)	14 (15%)	1 (3%)	10 (11%)	25 (71%)	68 (72%)	34 (97%)	84 (87%)

>12 months	29 (23%)		44 (34%)		29 (22%)		27 (21%)		27 (48%)	
	US	PLS	US	PLS	US	PLS	US	PLS	US	PLS
	13 (37%)	16 (17%)	10 (29%)	34 (36%)	6 (17%)	23 (25%)	6 (17%)	21 (22%)	29 (83%)	71 (76%)


*^∗^p < 0.0001; ^∗∗^Censored by death and kidney transplantation; US, urgent-start; PLS, planned-start.*

**Table 6:** Causes of death in patients with urgent- and planned PD start.

Time	Cause	US	PLS	Time	Cause	US	PLS
90 days	Cardiovascular	4	–	>12 months	Cardiovascular	1	6
	AIDS	–	1		Peritonitis	1	4
	Unknown	1	–		PAD	3	3
90 days–12 months	Cardiovascular	–	1		Malignancy	2	–
	Malignancy	1	–		Other/unknown	–	1


*PAD, peripheral arterial disease; US, urgent-start; PLS, planned-start.*

At the end of observation only 27 (21%) of all studied patients (17% from urgent- and 22% from planned-start group) stayed on PD. The most frequent reason for PD cessation was kidney transplantation – 34% of all patients (29 and 34%, respectively), death – 23% of all patients (37 and 17%, respectively), and transition to HD – 23% of all patients (17 and 25%, respectively). The causes of transition to HD are presented in Table 7.

**Table 7:** Causes of change to HD in patients with urgent- and planned PD start.

	Urgent-start (*n* = 35)		Planned-start (*n* = 94)	
Time	Patients	Causes	Patients	Causes
90 days	0		2 1	Loss of independence
			1	Patients decision
				Onco-surgery
90 days –	1	Peritonitis	2	Peritonitis
12 months			2	Loss of
			2	independence
				Leakage
>12 months	2	Membrane	5	Peritonitis
	1	failure	3	Loss of
	1	Peritonitis	3	independence
	1	Leakage Non-compliance	2	Membrane failure Abdominal surgery


Cox proportional analysis was performed for patient survival, method survival and both in 90 days, 1 year and all follow-up period. The results are presented in Table 8.

**Table 8:** Independent risk factors for patient and patient-method outcomes (Cox proportional analysis).

	90 days		12 months		Whole observation period	
Variable	Patient survival	Patient and technique survival^∗^	Patient survival	Patient and technique survival^∗^	Patient survival	Patient and technique survival^∗^
eGFR	1.0;	1.02;	0.84;	0.99;	1.0;	1.02;
	CI, 0.88–1.13;	CI, 0.94–1.11;	CI, 0.62–1.15;	CI, 0.84–1.17;	CI, 0.88–1.13;	CI, 0.9–1.11;
	NS	NS	NS	NS	NS	NS
Hb	0.8;	0.7;	1.46;	1.0;	0.77;	0.67;
	CI, 0.53–1.1;	CI, 0.48–1.04;	CI, 0.75–2.87;	CI, 0.67–1.52;	CI, 0.53–1.11;	CI, 0.51–0.87;
	NS	NS	NS	NS	NS	*p* = 0.003
CCI	1.6;	1.4;	1.4;	1.24;	1.43;	1.2;
	CI, 1.06–2.4;	CI, 1.09–1.78;	CI, 1.02–1.79;	CI, 1.05–1.47;	CI, 1.2–1.66;	CI, 1.08–1.32;
	*p* = 0.02	*p* = 0.007	*p* = 0.01	*p* = 0.02	*p* = 0.00002	*p* = 0.0003
Time ≤2 days	0.7;	8.53;	5.11;	4.29;	1.69;	1.4;
	CI, 0.13–3.65;	CI, 0.77–94.89	CI, 0.58–44.4;	CI, 0.73–25.1;	CI, 0.44–6.47;	CI, 0.56–3.51;
	NS	*p* = 0.02	*p* = 0.03	*p* = 0.04	*p* = 0.07	NS
Time >14 days	0.6;	0.18;	0.08;	0.38;	0.21;	0.56;
	CI, 0.16–1.86;	CI, 0.02–1.25;	CI, 0.004–1.67;	CI, 0.08–1.89;	CI, 0.08–0.58;	CI, 0.28–1.12;
	NS	*p* = 0.01	*p* = 0.04	*p* = 0.07	*p* = 0.0006	*p* = 0.02


*CCI, Charlson Comorbidity Index; Time, time between catheter insertion and PD start. ^∗^Censored by death and kidney transplantation.*

During the first 90 days of dialysis therapy the only important negative predictor for patient survival appeared to be CCI (HR 1.6 [CI: 1.06–2.4]; *p* = 0.02); however, later on, both CCI and time from catheter implantation to PD start were found to be significant. The same applies for both method and patient survival: CCI (HR 1.24 [CI: 1.05–1.47]; *p* = 0.02), time from catheter implantation (continuous variable HR 0.66 [CI: 0.48–0.91]; *p* = 0.01) and ≤ 2 days (HR 4.29 [CI: 0.73–25.1]; *p* = 0.04) were found to be predictive during the whole analyzed period, although after 1 year – time >14 days lost its predictive value (*p* = 0.07). There were no predictors for method survival alone in any analyzed period.

## Discussion

**FIGURE 1:**
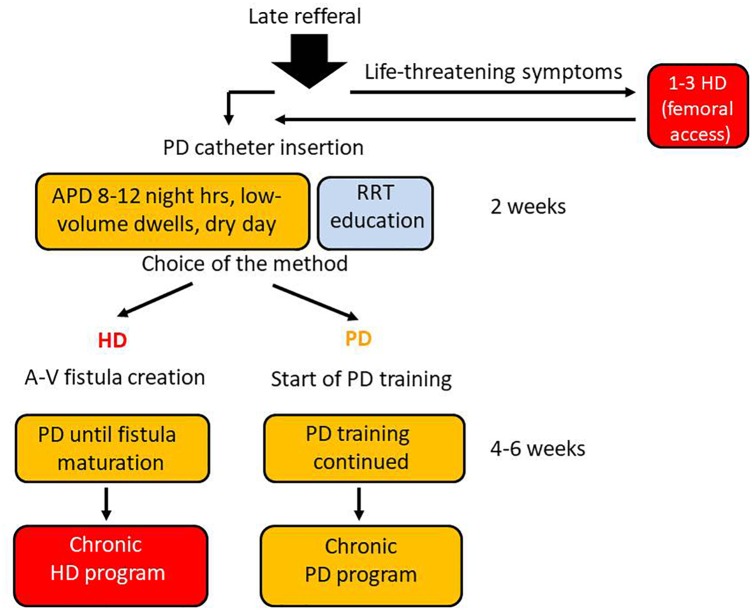
PD urgent–start program realized in our nephrology unit. Every late referral patient is quickly but carefully evaluated. Those who have no contradictions to PD and give an informed consent for PD as a bridge to final therapy choice are qualified to the program. The patients with life-threatening clinical symptoms like: pulmonary edema, severe hyperkalemia or acidosis, are given 1–3 short HD treatments via acute femoral catheter, before peritoneal access creation. The peritoneal catheter is introduced by a surgeon from our team, and the low-volume 8–12 night hours APD is started, with concomitant RRT education program for the patient and his family. If – after receiving sufficient information – the patients decide to remain on PD, the PD training is started, while in case when he/she considers HD as a more suitable RRT option – a arteriovenous fistula is created and PD continued until matured.

In our nephrology clinic, the PD urgent-start pathway, without typical 2-week break-in period, was opened 14 years ago; its outline is presented in Figure 1. It was meant for late-referral patients, able and prone to join PD-first program, with its main advantages such as: keeping the vascular system intact, preserving their residual renal function and retaining life-style flexibility. There was also our hope that early unplanned PD may be a good method to increase use this option of RRT. Finally, for patients who ultimately decided to change the treatment for HD, PD treatment continued until arteriovenous fistula maturation enabled to avoid temporary vascular access catheter placement. In this paper we compare the short- and long-term outcomes of consecutive 35 patients after urgent- and 94 patients after the planned start, who have commenced PD as a first RRT in our unit during this period.

The results confirm that PD can be a safe method of introducing RRT in unplanned acute dialysis settings. In general they are comparable with those of other authors, presented in Table 9 (Song et al., 2000; Banli et al., 2005; Povlsen and Ivarsen, 2006; Jo et al., 2007; Lobbedez et al., 2008; Yang et al., 2011; Casaretto et al., 2012; Ghaffari, 2012; Koch et al., 2012; Masseur et al., 2014; Alkatheeri et al., 2016; Bitencourt Dias et al. 2016, 2017; Jin et al., 2016; Pai et al., 2016; Wong et al., 2016; Xu et al., 2017; Wang et al., 2017; Nayak et al., 2018). However, there are many methodological differences among these studies concerning patients population, the technique of peritoneal catheter placement, or length of break-in period. In most of them the observation period was rather short (mostly 3–6 months), and only some of them have the control groups. In the presented study the median of the observation time was 19 months.

**Table 9:** Studies published during lasts years on urgent PD start.

Reference	Patients (groups)	Insertion technique	Urgent-start intervention	Observation period	Mechanical complications	Infectious complications	Survival (patient survival, method survival)
Povlsen and Ivarsen, 2006	140 pts (52-US vs. 88-PLS)	Surgical	APD < 24 h after catheter insertion	3 months	Leakage : 7.7% in US vs. 0% in PLS. Catheter dysfunction: 15% in US vs. 5.8% in PLS	Peritonitis: 15% in US vs. 15% in PLS ESI: 3.9% in US vs. 3.8% in PLS	Technique survival: 87% for US, 90% for PLS
Yang et al., 2011	310 pts (226-US vs. 84-PLS)	Surgical	CAPD 48–64 h after catheter insertion	6 months	Leakage: 2.2% in US vs. 2.4% in PLS Catheter dysfunction: 1,3% in US vs. 0% in PLS	Peritonitis: 4% in US vs. 2.4% in PLS ESI: 1.3% in US vs. 0% in PLS	Not specified
Pai et al., 2016	149 pts (80-US vs. 69-PLS)	Surgical	6–13 days after catheter insertion	30 ± 25 months	–	Peritonitis: 1/65 patient-months in US vs. 1/95 patient-months in PLS	Drop out of PD 45 in early and 34 in delayed starters
Jin et al., 2016	178 pts (96-US vs. 82-US HD)	Surgical	PD start within 14 days after catheter insertion		Catheter malposition 3.1%	Peritonitis: 2.1% ESI: 0%	3 months survival 98% for PD and HD 1 year survival 92% for PD, 93% for HD
Xu et al., 2017	922 pts US	Surgical	50% within 1 day after catheter insertion	Median 31 months	Abdominal wall complications 4.8% Catheter complications 9.5%	–	36% pts continued to receive PD therapy
Wang et al., 2017	101 pts US	Surgical	2 days after catheter insertion	12 months	Leakage 10% in IPD vs. 3.9% in APD Catheter malposition 4% in IPD vs. 3.9% in APD	Infection 26% in IPD and 13.7% in APD	–
Nayak et al., 2018	56 pts (32-US vs. 24 PLS)	Surgical	≤48 h after catheter insertion	90 days	Leakage 9.4% in US vs. 0% in PLS Catheter migration 25% in US vs. 16.7% in PLS	Peritonitis: 9.4%???	Technique survival 91% for US, 96% for PLS
Casaretto et al., 2012	11 pts US	Laparoscopic	APD < 48 h after catheter insertion	90 days	No leaks, catheter dysfunction in 1 patient	No peritonitis	–
Koch et al., 2012	123 pts (66-US vs. 57-US HD)	Laparoscopic	APD during 12 h after catheter implantation	4.7 ± 2.0 months	Catheter dysfunction: 7.6% for PD vs. 5.3% for HD	Bacteremia 3% for PD vs. 21% for HD. Peritonitis: 3%, ESI: 4.5%	6 months survival 70% for PD, 58% for HD (NS)
Masseur et al., 2014	81 pts US	Laparoscopic	ADP – immediately to 3–6 days after catheter implantation	3 months	No leaks Catheter dysfunction in eight patients	No peritonitis	95%
Alkatheeri et al., 2016	30 pts US	Laparoscopic or percutaneous	APD – immediately (6 pts) to median 6 days after catheter implantation	Median 201 days	Leakage in 10%; Catheter dysfunction in 20%	Peritonitis – 1 (1:319 patient-months) ESI 2 (1:159 patient-months)	3 months patient survival 100%; technique survival 93%
Song et al., 2000	59 pts US	Percutaneous	Immediately (<24 h)after catheter implantation: (I) gradual increase in exchange volume (II) Full exchange volume (2 L)	12 months	Leakage: 9.5% in Group I vs. 10.5% in Group II Catheter dysfunction: 4.8% in Group I vs. 5.3% in Group II	Peritonitis: 24% in Group I vs. 16% in Group II ESI: 9.5% in Group I vs. 5.3% in Group II	Catheter survival: Group I – 86%, Group II – 84%
Banli et al., 2005	41 pts US	Percutaneous	CAPD incremental since 6-th day after implantation	–	Leakage: 4.8%; Catheter dysfunction: 2.4%	Peritonitis: 2.4% ESI: 0%	–
Jo et al., 2007	51 pts US	Percutaneous	CAPD immediately after catheter implantation	12 months	Leakage: 2%; Catheter dysfunction: 12%	Peritonitis: 4% ESI: 4%	–
Ghaffari, 2012	27 pts (18-US vs. 9-PLS)	Percutaneous	Urgent: <2 weeks after catheter implantation Planned: 2–4 weeks after catheter implantation	3 months	Leakage: 33% in US vs. 11% in PLS Catheter dysfunction: 11% in US vs. 22% in PLS	Peritonitis: 1:110 patient-months in US vs. 1:42 patient-months in PLS ESI: 1:55 patient-months in US vs. 1:42 patient-months in PLS	–
Bitencourt Dias et al. 2016	76 pts (35-US vs. 6-PLS vs. 29-US HD vs. 6-PLS HD	Percutaneous	High volume PD < 48 h after catheter implantation	3 months	Leakage: 2.8% Catheter dysfunction: 20%	Peritonitis: 14.2% ESI: 8.6%	Patient survival – 80% Technique survival – 86%
Bitencourt Dias et al., 2017	51 pts US	Percutaneous	High volume PD < 72 h after catheter implantation	180 days	Leakage: 9.7% Catheter migration: 16%	Peritonitis: 0.5 patient/y ESI 17%	Patient survival – 82% Technique survival – 86%
Wong et al., 2016	81 pts US	Surgical or laparoscopy or percutaneous	Emergent – within 48 h of catheter implantation; Urgent – 48 h – 14 days after catheter implantation	12 months	Leakage: 5% Catheter dysfunction: 15%	Peritonitis – 16% (72/100 patient-years)	–
Lobbedez et al., 2008	60 pts (34 US PD vs. 26 US HD)	Not specified	APD 9.6 ± 10.3 days (median: 4 days) after catheter insertion	12 months	Leakage: 5.8%	The survival free of peritonitis: 73% at 6 months and 55% at 1 year	Patient survival: 83% for PD, 79% for HD PD technique survival – 90% after 6 months and 88% after 12 months


The biggest concern about acute PD start is the risk of early mechanical complications: dialysate leaks as well as the catheter dysfunction. It may be that an early rise in peritoneal pressure negatively affects the wound healing and facilitates leakage. This can be further intensified by hypoalbuminemia, relatively often present in ESRD patients who need urgent dialysis start. In the presented study a short break-in period together with low serum albumin occurred to be the independent predictors for early dialysate leakage. The risk of this complication in patients who started PD urgently was 11% (vs. 0% in planned start group, *p* < 0.001), and its overall incidence was higher than described by some other authors (Povlsen and Ivarsen, 2006; Lobbedez et al., 2008; Sharma et al., 2008; Yang et al., 2011; Liu et al., 2014). There was also a higher incidence of bleeding into peritoneal cavity in urgent PD start group. However, after several days of peritoneal rest PD was resumed, and – unlike in some other studies – none of the patients needed a surgical intervention, and PD technique success at 3 months as well as during the whole observation was similar in both groups. The rates of late non-infectious complications were in both groups similar, with an exception of PD catheter migration, more frequently seen in the planned-start group. It did not affect the technique and patient survival either.

Contrary to the common perception that early use of peritoneal catheter may increase a risk of infectious complications such as exit-site/tunnel infections and peritonitis, we did not observe any of these in the early 4-week period of the treatment. The incidence rates during the whole observation period were in both studied groups similar. A shorter time to first peritonitis episode in urgent-start group shorter (*p* = 0.04) seems to be rather a consequence of a shorter observation period in this group. In the regression logistic analysis none of analyzed parameters had influence on peritonitis or exit-site/tunnel infection occurrence.

We found significantly higher mortality rate after first 90 days of the therapy in patients initiating PD urgently (14 vs. 1.1%, *p* < 0.0001). Although both groups were comparable in respect to age, many other factors may explain the reduced short-term survival in the unplanned starters (Lobbedez et al., 2008; Ivarsen and Povlsen, 2014). It is well-known that first 90 days of any dialysis therapy is a period of disproportionately high mortality and that in patients who start the treatment urgently and are often in a challenging clinical condition the outcomes are worst, with the percentages reaching even 30% in unplanned HD (where a part of which may be attributable to CVC (Khan et al., 1995; Collins et al., 2009; Mendelssohn et al., 2009; Baer et al., 2010; Panochia et al., 2016). Our urgent start patients were “sicker”: had more advanced uremia, lower albumin and hemoglobin levels and worse general clinical status, with necessity of dialysis within first 48 h in 51% of them, including 3 (8.6%) patients with life-threatening uremic symptoms in whom 2–3 short emergency HD via femoral access were performed before PD catheter placement.

The higher mortality rate in the urgent-start group persisted after 1 year (17 vs. 2%) and at the end of observation (37 vs. 17%), although the difference did not reach the statistical significance, possibly because small numbers of studied patients. The only significant predictor of patient death during first 90 days of dialysis therapy appeared to be CCI (HR 1.6 [CI: 1.06–2.4]; *p* = 0.02), however, later on both: CCI and time from catheter implantation to PD start were found to be significant.

The studied group consists of unselected incident patients started PD in our unit and seems to be fairly typical patients population treated with PD (age, comorbidity, ∼30% urgent start). The mortality rate in this group in the entire observation period (mean: 25.6 ± 23.8, median: 19 months) was 23%, and 10.6% in the first year (4.6%–90 days, 6%–90 days–12 months). It seems to be comparable with reported mortality rates in dialysis populations (USRDS, ERA-EDTA Registry), which despite some improvement in the last decade, remains very high (∼20%) with a universal phenomenon of increased mortality early after dialysis initiation (Robinson et al., 2014). The mortality rate was distinctly, however not statistically significant higher (effect of the sample size?) in urgent-starters in all time intervals, and this is good established observation that urgent dialysis start is an important factor of poor prognosis (short and long-term) irrespective of dialysis modality and generally results from various aspects related to uremic complications and comorbidities, as well as dialysis issues (i.e., CVC in HD patients). In our study, in Cox proportional analysis the time ≤ 2 days from catheter implantation to dialysis start (which may be a marker of necessity of dialysis initiation – more advanced uremia, worse general clinical status), and CCI proved to be an independent risk factors for patient survival in the whole observation.

With death and renal transplantation being the censored events, the PD technique survival was excellent in both, urgent and planned start groups, being respectively: 100 and 96% at 3 months, 97 and 87% at 12 months, and 83 and 76% at the end of the observation. These percentages are higher than reported by the other experienced urgent-start PD centers. In a French study by Lobbedez et al. (2008), which included 34 unplanned dialysis patients the actuarial PD technique survival was 90% at 6 months and 88% at 1 year. In the study done by Povlsen and Ivarsen, who compared outcomes of 52 urgent start with 52 planned-start patients, the 3-month PD technique censored survival rates were even lower, with corresponding values: 87 and 90% (Povlsen and Ivarsen, 2006).

## Conclusion

Peritoneal dialysis may be a feasible and safe alternative to HD in patients who need to start dialysis urgently without established dialysis access, with an acceptable complications rates, as well as patient and technique survival. We found this method an important part of full RRT program in every reference dialysis unit, allowing for real free individual choice of the modality. However to be successful PD urgent start program needs adequate infrastructure, expertise, good organization with continuous availability of experienced surgeons or other doctors who place peritoneal catheters, dedication of the complete team, and a good RRT educational program, adjusted to the unplanned setting, to give the full possibility of unbiased final choice of the RRT method, that fits best to their lifestyle.

## Ethics Statement

The study was exempted from the requirement of the Ethics Committee, as all the patients were in end-stage kidney disease and require renal replacement therapy in other planned or urgent manner. All of them singed the informed consent to start renal replacement therapy as required by the National Health found (routine informed consent for the procedure of increased risk).

## Author Contributions

EW, AG, and JM-R conceived the idea for the study and contributed to the design of the research. EW and JM-R performed the statistical analysis of the collected data and performed the analysis. EW, JM, and JM-R were involved in the preparation of the manuscript. EW, AG, and KG were involved in data collection. All the authors analyzed the data, edited, and approved the final version of the manuscript.

## Conflict of Interest Statement

The authors declare that the research was conducted in the absence of any commercial or financial relationships that could be construed as a potential conflict of interest.
